# A Highly Sensitive Humidity Sensor Based on Ultrahigh-Frequency Microelectromechanical Resonator Coated with Nano-Assembled Polyelectrolyte Thin Films

**DOI:** 10.3390/mi8040116

**Published:** 2017-04-05

**Authors:** Wenpeng Liu, Hemi Qu, Jizhou Hu, Wei Pang, Hao Zhang, Xuexin Duan

**Affiliations:** State Key Laboratory of Precision Measuring Technology & Instruments, Tianjin University, Tianjin 300072, China; liuwenpeng@tju.edu.cn (W.L.); mrhjz1222@tju.edu.cn (J.H.); weipang@tju.edu.cn (W.P.); haozhang@tju.edu.cn (H.Z.)

**Keywords:** humidity sensor, film bulk acoustic resonator (FBAR), ultrahigh frequency, microelectromechanical resonator, nano-assembled polyelectrolyte (PET) thin films, wireless sensor networks (WSNs)

## Abstract

We developed a highly sensitive humidity sensor based on the combination of ultrahigh-frequency film bulk acoustic resonator (FBAR) and nano-assembled polyelectrolyte (PET) thin films. The water molecule absorption efficiency was optimized by forming loosely-packed PET nanostructures. Then, the humidity sensing characteristics were analyzed in terms of sensitivity, linearity, reversibility, stability and detection limit. As a result, PET-coated FBAR exhibits excellent humidity sensitivity of 2202.20 Hz/ppm, which is five orders of magnitude higher than quartz crystal microbalance (QCM). Additionally, temperature dependence was investigated with the result that PET-coated FBAR possessed a higher sensitivity at low temperature. Furthermore, we realized the selective detection of water vapor from volatile organic compounds (VOCs) with respect to the polarity property. Owing to the high sensitivity, miniaturized size and ultrahigh operating frequency, PET-coated FBAR is uniquely favorable as a wireless humidity sensor node to integrate into wireless sensor networks (WSNs).

## 1. Introduction

Monitoring of environmental humidity is crucial in fields ranging from meteorology, agriculture and industry to medicine/healthcare development and food science [1,2,3]. Based on this need, many types of humidity sensors based on different transducing techniques have been developed. Among them, capacitive and resistive humidity sensors have been dominating the markets over the years [4,5]. Other approaches, including surface acoustic waves (SAW) [6], quartz crystal microbalance (QCM) [7], field effect transistor (FET) [8], and fluorescent emission [9], have also been adopted to monitor humidity with success. Recently, the trend towards the implementation of wireless sensor networks (WSNs) [10], in which the spatially-distributed miniaturized sensors monitor physical or environmental conditions through wireless communication functionalities, requires sensors be small and consume less energy. As one type of microelectromechanical resonators, film bulk acoustic resonator (FBAR) has emerged to meet the trend [11,12]. FBAR usually operates at two orders of magnitude higher frequency than QCM and is cable of detecting small mass changes at even pictogram order. Hence, it has been extensively employed for fundamental science and practical engineering [13,14]. It features a greatly reduced dimension of the sensing element, which is beneficial to integrate into miniaturized platform and obtain lab-on-a-chip. Also, the compatibility with conventional complementary metal oxide semiconductor (CMOS) technology promotes the mass production of FBAR with low cost. All these advantages make FBAR a good candidate as humidity sensor to integrate into WSNs in the development of internet of things. Nevertheless, the FBAR-based humidity sensor reported in the literature suffers from the poor sensing characteristics with respect to the sensitivity, detection limit and stability [11,12,15].

As a crucial component of surface-based sensors, the sensitive layer functionalized on the sensing area enhances the sensitivity by providing more absorption sites for analytes. Nowadays, materials with nanostructures such as nanofiber [16], nanowire [17], nanopore [18], and nanotube [19], have been adopted as humidity-sensitive layers to improve the humidity sensing characteristics. Owing to the large surface area, these nanostructured materials provide tremendous absorption sites for water molecules to enhance the surface activity. Among them, nanostructured polyelectrolytes are of particular interest due to the flexibility, low cost and long-term stability. Molecular surface self-assembly is a unique approach for the formation of nanostructured polyelectrolyte (PET) thin films with controlled thickness at nanometer range [20,21]. It is based on alternate exposure of charged substrate to PET solutions with oppositely charged polycations and polyanions, which enable electrostatically driven self-assembly of these PETs [22]. In a wide variety of research fields, molecular surface self-assembly is one of the most prominent methods to build ultrathin PET films, with many distinct advantages over other methods, including simplicity, controllable thickness and no requirements of complex equipment.

In this work, we are motivated to develop a highly sensitive humidity sensor using ultrahigh-frequency FBAR coated with nano-assembled PET thin films. The water vapor absorption behavior of different morphology of PET thin films was discussed. Then, the sensing characteristics including linearity, sensitivity, stability, reversibility, selectivity and detection limit were evaluated. Temperature dependence for humidity measurement was investigated in order to ensure the performance within a wide range of applications. We also analyzed the selective absorption of water molecules from volatile organic compounds (VOCs). Owing to the high sensitivity, miniaturized size and ultrahigh operating frequency, FBAR coated with nano-assembled PET thin films is a potential candidate as a wireless humidity sensor to realize the integration with WSNs.

## 2. Materials and Methods

### 2.1. Materials

Poly(sodium 4-styrenesulfonate) (PSS, *M*_w_ = 70,000), poly(diallyldimethytlammonium choride) (PDDA, *M*_w_ < 10,000), poly(acrylic acid) (PAA, *M*_w_ = 160,000) and poly(allylamine hydrochloride) (PAH, *M*_w_ = 58,000), were purchased from Sigma Aldrich (St. Louis, MO, USA). (3-Aminopropyl)triethoxysilane (APTES) were purchased from Aladdin Industrial Corporation (Shanghai, China). All chemicals were used without further purification. N-propyl alcohol (NPA) and n-hexane were purchased from Real & Lead Chemical Co. Ltd. (Tianjin, China). PSS/PDDA solutions were prepared by dissolving them separately into ultrapure water, and ultrasonicated for 15 min.

### 2.2. Experimental Setup

**Figure 1:**
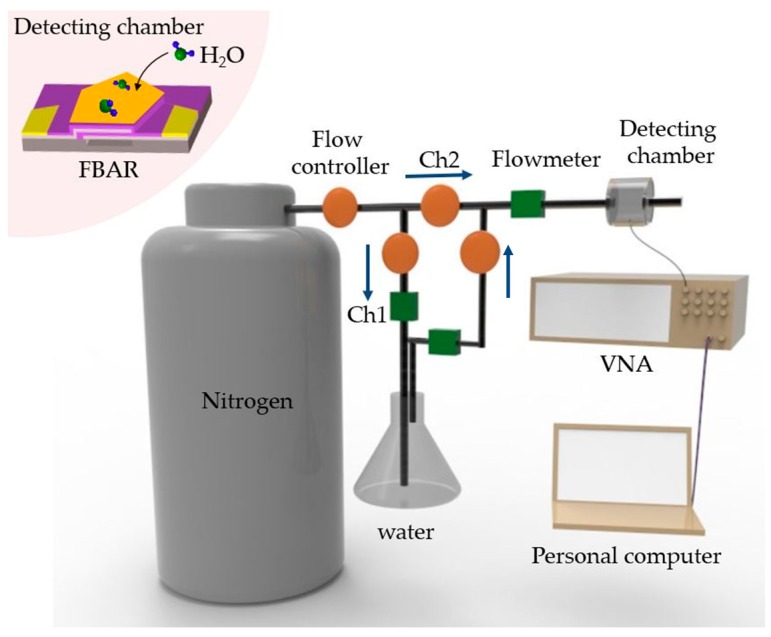
Cartoon showing the experimental setup for humidity sensing using polyelectrolyte (PET)-coated film bulk acoustic resonator (FBAR).

Figure 1 depicts the schematic diagram of the experimental setup. A customized gas delivery system was designed to deliver water vapor at a constant temperature of 28 °C. In the system, a channel (Ch1) was used to bubble out the water vapor through liquid phase by nitrogen gas (99.999%), while another channel (Ch2) filled with nitrogen gas was employed as a dilution line to adjust the relative concentration of final mixed gas by changing the ratio of two channel flow rates via flow controllers. Before each experiment, a commercial humidity sensor was introduced to calibrate the volume concentration of water vapor inside the detecting chamber. FBAR coated with nano-assembled PET thin films was mounted onto an evaluation board, and then connected to a computer-controlled vector network analyzer (VNA, B5071C, Keysight, Santa Rosa, CA, USA) to simultaneously record the frequency shift.

### 2.3. Device Fabrication

**Figure 2:**
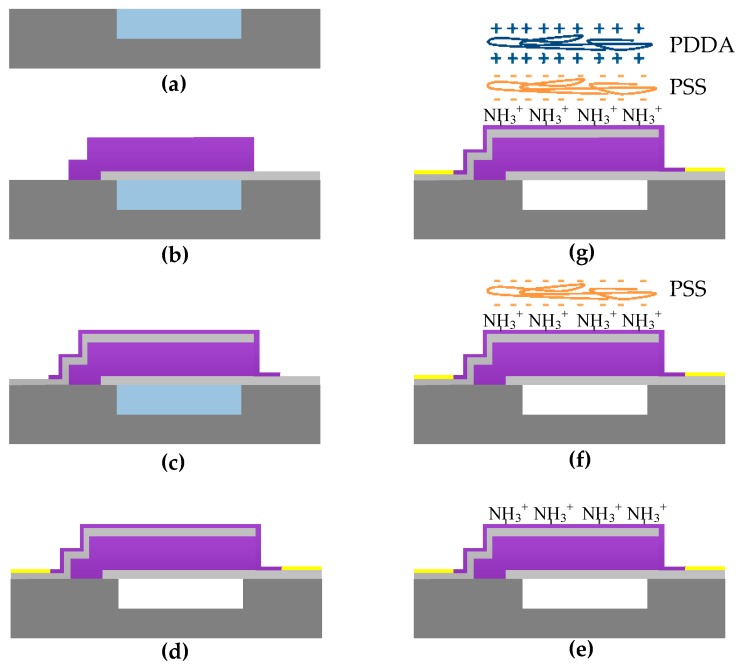
Schematic illustrating the fabrication process of FBAR and molecular surface self-assembly of PETs. (**a**) Etching of air cavity and deposition of phosphosilicate glass (PSG); (**b**) deposition of bottom electrode and piezoelectric layer; (**c**) deposition of top electrode and passivation layer; (**d**) deposition of Au pads and release of PSG; (**e**) amino-silanization of FBAR; (**f**) nano-assembly of poly(sodium 4-styrenesulfonate) (PSS); (**g**) nano-assembly of poly(diallyldimethytlammonium choride) (PDDA).

FBAR comprises a piezoelectric layer sandwiched by the top electrode and bottom electrode. It is fabricated through standard semiconductor manufacturing technology, as shown in Figure 2a–d. Briefly, an air cavity was first created on the silicon substrate by reactive ion etching, and filled with phosphosilicate glass (PSG) as sacrificial layer by chemical vapor deposition. A 600-nm molybdenum (Mo) and 800-nm high-quality c-axis oriented aluminium nitride (AlN) were sequentially deposited by radio frequency (RF) sputter and patterned to create the bottom electrode and piezoelectric layer. Another 600 nm Mo and 400 nm AlN were sequentially deposited and patterned as the top electrode and passivation layer. A 10 nm/100 nm Cr/Au composite film was then deposited by physical vapor deposition and patterned to form the pads. Finally, PSG was etched by diluted hydrofluoric acid to completely release the device.

### 2.4. Molecular Surface Self-Assembly of Polyelectrolytes (PETs)

As shown in Figure 2e–g, FBAR was first exposed to the air plasma for 5 min to form hydroxyl groups on a passivation layer [23], followed by amino-silanization with APTES in a chemical gas deposition system (Yield Engineering Systems, Inc., Livermore, CA, USA). Then we adopted both dipping-assisted and spinning-assisted methods to realize the molecular surface self-assembly of PETs in a layer-by-layer fashion. For the dipping-assisted method, NH_2_-functionalized FBAR was immerged into PSS solution for 2 min and rinsed by ultrapure water for 30 s. The PSS-coated FBAR was then dipped into PDDA solution with the same procedure, resulting in the first PSS/PDDA bilayer. PET thin films with various thickness were accomplished by tuning the number of PSS/PDDA bilayers. For multiple bilayers assembly, a homemade fully automatic dipping robot was used to maintain the uniformity of the nano-assembled PET thin films. For the spinning-assisted method, NH_2_-functionalized FBAR was fixed onto a spin-coating machine, the spinning speed was set at 2000 rpm. PSS/PDDA solutions were alternately pumped onto FBAR via syringes by 200 μL/s for 5 s, followed by spin-drying for 15 s. An ellipsometer was used to record the thickness of nano-assembled PET thin films.

## 3. Results and Discussion

### 3.1. Characterization of FBAR

**Figure 3:**
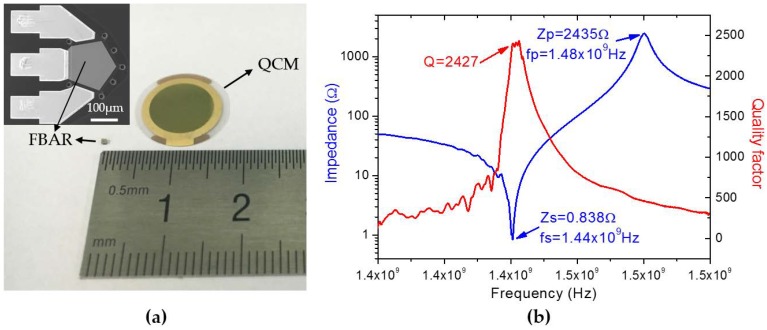
FBAR characterization. (**a**) A comparison of the size between FBAR and quartz crystal microbalance (QCM). The top-left inset shows the top-view scanning electron microscopy (SEM) image of FBAR; (**b**) magnitude of impedance and *Q* value over frequency.

The fabrication process of FBAR is compatible with standard wafer-scale semiconductor technology, hence FBAR possesses competitive advantages for miniaturization and mass production with low cost, which is particularly important for integration as wireless sensor node into WSNs. Figure 3a shows the comparison of the size between FBAR and QCM, indicating significantly smaller footprints of FBAR. The top-left inset of Figure 3a shows the top-view scanning electron microscopy (SEM) image of FBAR with the pentagonal sensing area of 20,000 μm^2^. This pentagonal sensing area is defined by the top electrode with an AlN passivation layer. The passivation layer protects top electrode from vapor and thus improves device stability. Additionally, selective amino-silanization on the passivation layer ensures the selective assembly of PET thin films on sensing area without contaminating Au pads. 

Figure 3b presents the magnitude of impedance over frequency, as well as the quality factor which is supposed to be an indicator of energy losses in the system [24]. The impedance at series resonant frequency ($f_{s}$ = 1.44 × 10^9^ Hz) and parallel resonant frequency ($f_{p}$ = 1.48 × 10^9^ Hz) is 0.838 Ω and 2435 Ω respectively. The quality factor is as high as 2427 when operating at ultrahigh frequency of 1.44 × 10^9^ Hz, indicating high acoustic energy density and well-trapped waves within the piezoelectric layer. In addition, according to Sauerbrey’s equation [25], the frequency shift of mass-sensitive FBAR for a certain mass load increases proportional to the resonance frequency squared; hence, ultrahigh-frequency FBAR shows markedly enhanced sensitivity for humidity sensing.

### 3.2. Morphology Optimization of Nano-Assembled PET Thin Films

Nano-assembled PET thin films provide abundant absorption sites for water molecules by enlarging the surface area, leading to an enhanced sensitivity. Therefore, efforts are generally put into the optimization of the nanostructure of PET thin films to obtain excellent sensing performance. Here, we prepared the nano-assembled PET thin films with different morphology and investigated the morphology dependence of humidity sensing performance. The morphology can be tuned by choosing assembly methods and varying PET concentration. Thus, three types of PET thin films were assembled on FBAR surface at three conditions, which are summarized in Table 1.

**Figure 4:**
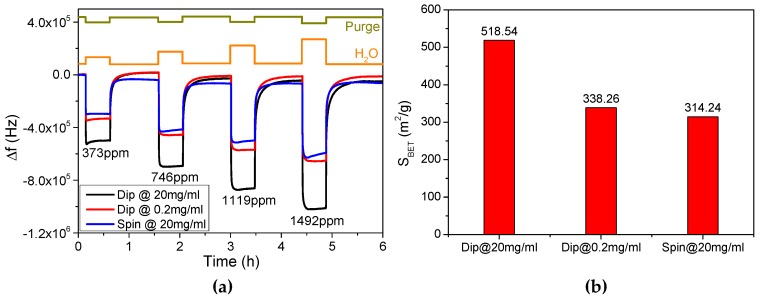
The morphology dependence of humidity sensing via PET-coated FBAR. (**a**) Real-time responses of FBARs with the volume concentration of water vapor changing from 373 ppm to 1492 ppm. Nano-assembled PET thin films were fabricated via dipping-assisted method in 20 mg/mL solutions (black curve), dipping-assisted method in 0.2 mg/mL solutions (red curve) and spinning-assisted method in 20 mg/mL solutions (blue curve); (**b**) specific surface area of nano-assembled PET thin films.

**Figure 5:**
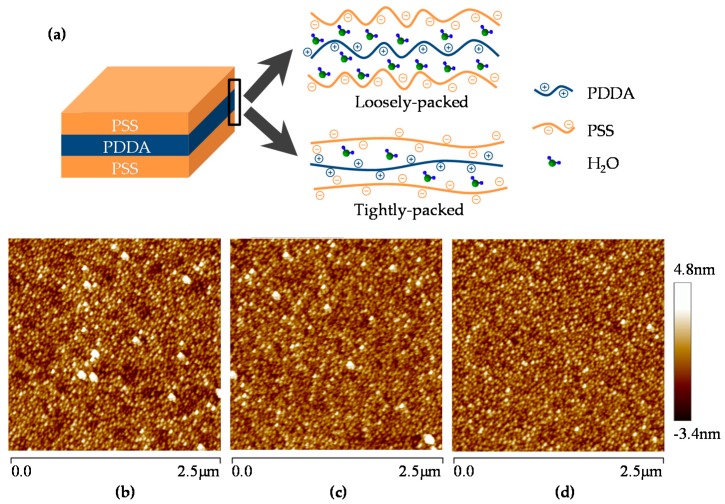
Comparison of loosely-packed and tightly-packed nanostructure of PET thin films. (**a**) Schematic illustration of the loosely-packed and tightly-packed nanostructure of poly(sodium 4-styrenesulfonate)/poly(diallyldimethytlammonium choride) (PSS/PDDA) bilayers; (**b**) atomic-force microscopy (AFM) height image showing the morphology of PSS/PDDA bilayers assembled via dipping-assisted method in 20 mg/mL solutions. The root-mean-square roughness is 1.09 nm ± 0.06 nm; (**c**) AFM height image showing the morphology of PSS/PDDA bilayers assembled via dipping-assisted method in 0.2 mg/mL solutions. The root-mean-square roughness is 0.95 nm ± 0.03 nm; (**d**) AFM height image showing the morphology of PSS/PDDA bilayers assembled via spinning-assisted method in 20 mg/mL solutions. The root-mean-square roughness is 0.90 nm ± 0.03 nm. The root-mean-square roughness was obtained from three different scanning areas for each sample.

We first analyzed the concentration dependence of PET thin films’ morphology and their water molecule absorption efficiency. Figure 4a shows the responses of FBARs for humidity measurement. Obviously, responses of PET thin films assembled in concentrated solutions (20 mg/mL) are higher than in dilute solutions (0.2 mg/mL). Such discrepancy can be explained by the different conformation of PET chains in different concentration. During the assembly process, PET chains interdigitate and form complexes with oppositely charged ones which have been formerly absorbed onto the surface, resulting in a certain internal roughness of PET thin films [26]. In concentrated solutions, the conformation of PET chains is less extended than in dilute solutions [27], leading to a higher internal roughness and loosely-packed nanostructure, as shown in Figure 5a.

The Brunauer–Emmett–Teller (BET) equation is introduced to characterize the nanostructure of PET thin films with respect to the specific surface area ($S_{\mathrm{BET}}$) [18,28]:

(1) $$\frac{1}{\nu \left(\frac{p_{0}}{p}-1\right)}=\frac{c-1}{\nu_{m}c}\times \frac{p}{p_{0}}+\frac{1}{\nu_{m}c}$$

(2) $$S_{\mathrm{BET}}=\frac{\nu_{m}Ns}{Va}$$

where p represents the partial vapor pressure and $p_{0}$ represents the saturated vapor pressure, ν is the absorbed mass of vapor, $\nu_{m}$ is the monolayer absorbed mass of vapor, c is the BET constant, N is Avogadro’s number, s is the adsorption cross section of the absorbed species, V is the molar volume of the vapor, and a is the mass of the sensitive layer. As a consequence, the PET thin films fabricated in 20 mg/mL solutions possess a larger specific surface area of 518.54 m^2^/g than in 0.2 mg/mL solutions whose specific surface area is 338.26 m^2^/g, as illustrated in Figure 4b and Supplementary Figure S1. Therefore, the loosely-packed nanostructure with larger specific surface area absorbs more water molecules and results in higher responses. Figure 5b,c present the morphology of PET thin films nano-assembled in concentrated and dilute solutions respectively. PET thin films fabricated in 20 mg/mL solutions are less uniform than in 0.2 mg/mL solutions. Quantitatively, the root-mean-square roughness of PSS/PDDA bilayers fabricated in 20 mg/mL solutions is 1.09 nm ± 0.06 nm, while it decreases to 0.95 nm ± 0.03 nm in 0.2 mg/mL solutions. This is in consistence with the BET analysis.

Similar results can be acquired from the comparative analysis between different assembly methods. As illustrated in Table 1, dipping-assisted and spinning-assisted methods were adopted to tune the morphology of PET thin films in 20 mg/mL solutions. Figure 4a,b present the responses of FBAR and BET fitting results for humidity measurement respectively. Responses of PET thin films assembled via dipping-assisted method are higher than via spinning-assisted method, and the specific surface area of spinning-assisted PET thin films is 314.24 m^2^/g, smaller than that of dipping-assisted ones. The small specific surface area of spinning-assisted PET thin films could be explained by the air shear force driven by the spinning process [29]. The air shear force extends the conformation of PET chains, leading to a low internal roughness and tightly-packed nanostructure. Figure 5d presents the morphology of spinning-assisted PET thin films assembled in 20 mg/mL solutions. The films feature the smoothest surface with the root-mean-square roughness of 0.90 nm ± 0.03 nm.

**Figure 6:**
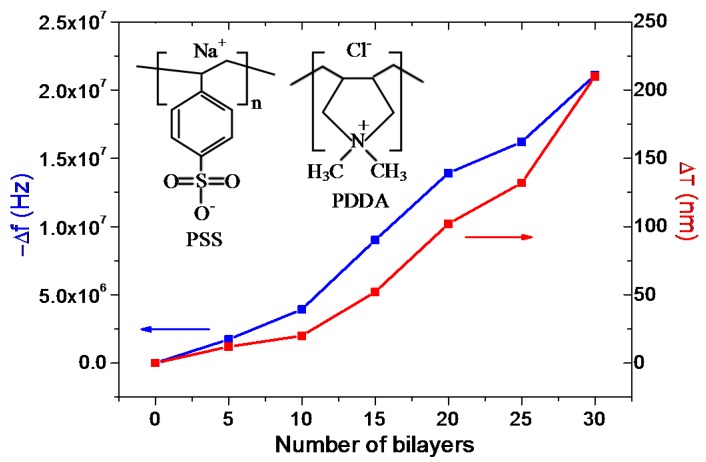
Frequency shift of FBAR and thickness growth of PSS/PDDA bilayers during the molecular surface self-assembly on NH_2_-functionalized FBAR.

For the following humidity measurements, PSS/PDDA films were assembled in 20 mg/mL solutions via the dipping-assisted method in order to obtain a large specific surface area and improve the sensitivity. Since PET thin films are not rigid films, excessive assembly of PSS/PDDA bilayers on FBAR surface leads to the dissipation of acoustic energy from the piezoelectric layer, which causes the loss of quality factor, resulting in the consequent decrease of sensitivity. Additionally, the classic Sauerbrey equation is valid only when the maximum mass load of PET thin films does not exceed two percent of the resonant frequency [23]. Therefore, we controlled the frequency shift resulting from the PET assembly within 1.44 × 10^9^ Hz × 2% = 2.88 × 10^7^ Hz. Figure 6 displays the frequency shift of FBAR and the thickness growth of PSS/PDDA films during the molecular surface assembly on FBAR surface. The PSS/PDDA films grow in an approximately exponential behavior with controlled thickness at nanometer range. After 30 assembly cycles, the thickness of PSS/PDDA films extends to 210 nm and the resonant frequency of FBAR decreases by 2.1 × 10^7^ Hz.

### 3.3. Humidity Sensing Characteristics

#### 3.3.1. Sensitivity and Linearity

**Figure 7:**
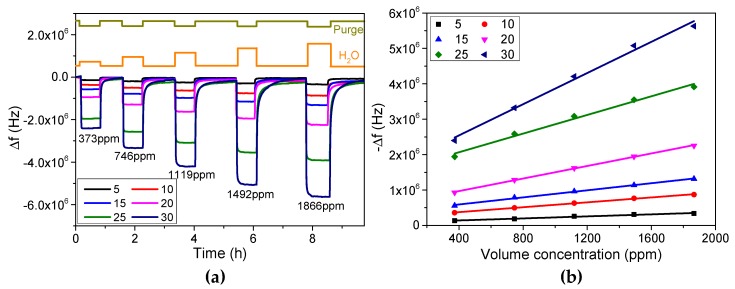
Humidity sensing via PET-coated FBAR. (**a**) Real-time responses of FBARs with the volume concentration of water vapor changing from 373 ppm to 1866 ppm. The number of PSS/PDDA bilayers ranges from 5 to 30; (**b**) frequency shift with respect to the various volume concentration of water vapor and number of PSS/PDDA bilayers.

In order to measure the humidity and investigate the sensitivity of FBAR, we prepared six FBARs coated with different numbers of PSS/PDDA bilayers. Figure 7a shows the real-time responses of FBARs as a function of volume concentration of water vapor ranging from 373 ppm to 1866 ppm. Obviously, FBARs present a fast response time for humidity measurement. With the growing number of PSS/PDDA bilayers and volume concentration, responses of FBARs increase gradually, which indicates that more water molecules are absorbed into the PET thin films. Figure 7b and Table 2 present the calibration curves and corresponding sensitivity (defined as the calibration curve slope with the unit of Hz/ppm), as well as the linearity (defined as *R*-Square) respectively. As a result, the frequency shift presents an excellent linearity within the volume concentration ranging from 373 ppm to 1866 ppm. The sensitivity increases from 143.61 Hz/ppm to 2202.20 Hz/ppm with the growing thickness of PET thin films, which is five orders of magnitude higher than a typical QCM [30,31]. The markedly enhanced sensitivity shows a great advantage for humidity measurement.

#### 3.3.2. Reversibility

It is worth mentioning that the resonant frequency recovers to initial level after adjusting the volume concentration of water vapor back to 0 ppm, indicating the reversible absorption behavior of the nano-assembled PET thin films. The reversible sensing of humidity sensors allows accurate calibration prior to humidity measurement. Additionally, it is very attractive from economical point of view by reusing the device. Therefore, FBAR coated with nano-assembled PET thin films represents a promising candidate for the development of regenerative electronic humidity sensors.

Additionally, it is clear that responses take a longer time to recover to initial level after exposing to higher volume concentration of water vapor. During desorption at low volume concentration, the large interspaces in PET thin films make water molecules pass through the materials easily. This contributes to the fast recovery of the humidity sensor. At high volume concentration, more water molecules are absorbed into the PET thin films and PET thin films are gelled. Thus, the interspaces are shortened, hindering desorption of water molecules from PET thin films. Therefore, longer purging time is required for recovering the sensor to initial level at higher humidity level. 

#### 3.3.3. Detection Limit, Noise and Stability

**Figure 8:**
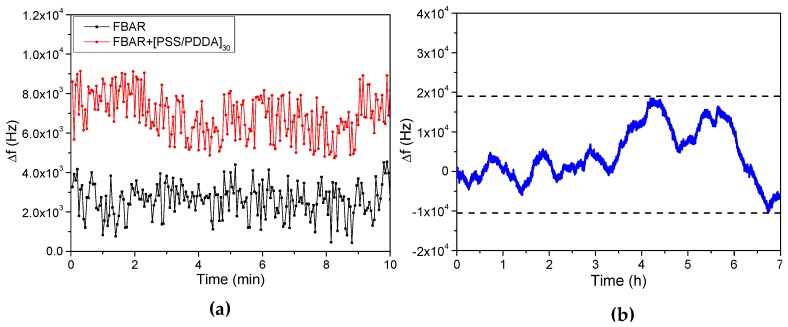
Noise and stability measurements of FBAR at ambient conditions. (**a**) Noise of both FBAR without coating and FBAR coated with 30 PSS/PDDA bilayers; (**b**) frequency drift of FBAR coated with 30 PSS/PDDA bilayers.

Considering that the noise level exhibited by PET-coated FBAR determines the theoretical detection limit for humidity sensing, we measured the noise for both FBAR without coating and FBAR coated with 30 PSS/PDDA bilayers at ambient conditions for 10 min, as shown in Figure 8a. The noise level (defined as the deviation between the first deciles and the nine deciles of frequency shift) for uncoated FBAR is 2.32 × 10^3^ Hz, while the noise level for FBAR coated with 30 PSS/PDDA bilayers is 3.20 × 10^3^ Hz. The slight increase of noise level after PET assembly origins from the inhomogeneity of PSS/PDDA bilayers which shows negative effect on the acoustic wave propagation [32]. Meanwhile, assuming the sensitivity of 2202.20 Hz/ppm and the signal-to-noise ratio of 3, the theoretical detection limit of FBAR coated with 30 PSS/PDDA bilayers for humidity is estimated to be as low as 43.64 ppm [33].

We also conducted the stability measurement for PET-coated FBAR. Figure 8b shows the frequency drift of FBAR coated with 30 PSS/PDDA bilayers after exposure in air for seven hours. As shown, the maximum frequency drift is limited to 3 × 10^4^ Hz during testing, much less than the frequency shift for the humidity measurement. This indicates the high stability of FBAR-based humidity sensor.

### 3.4. Temperature Dependence

**Figure 9:**
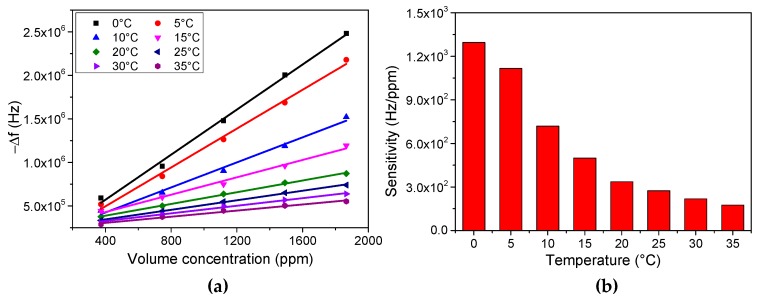
Temperature dependence of PET-coated FBAR for humidity sensing. (**a**) Frequency shift with respect to both the environmental temperature ranging from 0 °C to 35 °C and the volume concentration of water vapor changing from 373 ppm to 1866 ppm; (**b**) sensitivity of PET-coated FBAR with the environmental temperature changing from 0 °C to 35 °C.

It is known that the sensitivity of sensors is influenced by the environmental temperature [34,35,36]. In order to ensure that PET-coated FBAR is be able to perform humidity measurement within a wide range of wireless applications, we evaluate the sensor sensitivity at different temperature ranging from 0 °C to 35 °C, as shown in Figure 9. It is clear that responses of PET-coated FBAR vary with the environmental temperature. The sensitivity increases gradually at low temperature and the maximum sensitivity to humidity obtained is 1295.11 Hz/ppm at 0 °C, more than seven times higher than at 35 °C. The enhancement of sensitivity is due to the high partial partition coefficient in absorption equilibrium at low temperature [37,38]. In a specific water vapor concentration, the frequency of FBAR is stable when the absorption and desorption of water molecules in PET thin films reach a dynamic equilibrium. As the environmental temperature decreases, the water molecules possess less kinetic energy. This hinders the escape of water molecules from PET thin films and increases partial partition coefficient, as well as the amount of water molecules in PET thin films. Therefore, cooling device is preferred to further enhance the sensor sensitivity for ultralow humidity measurement. Results suggest that normalization with respect to the environmental temperature is needed for PET-coated FBAR for practical applications in humidity measurement. 

### 3.5. Selectivity Analysis

The selectivity of sensors shows the capability to discriminate the analytes of interest from a particular interference. In practice, the selectivity of gas sensors is usually achieved by the functionalization of sensing area with sensitive layer [39]. In our experiments, the selectivity of PET-coated FBAR was analyzed by introducing VOCs as interference.

**Figure 10:**
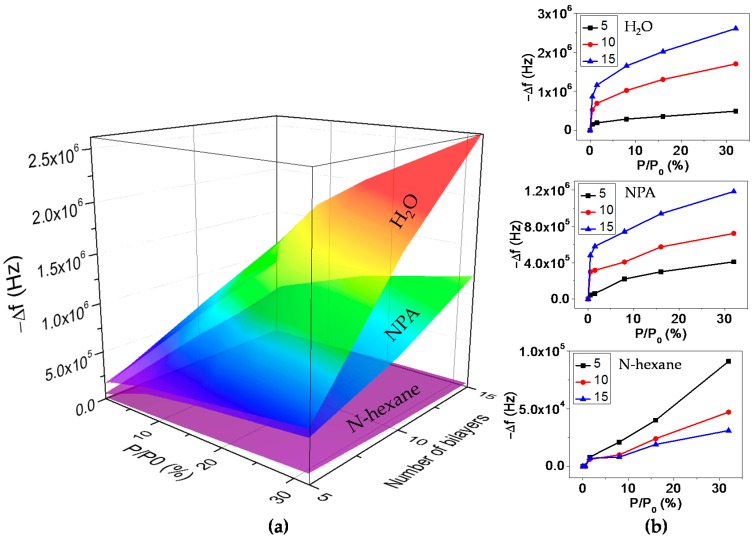
Frequency shift for the measurement of water, n-propyl alcohol (NPA) and n-hexane vapors. FBARs were coated with 5, 10 and 15 PSS/PDDA bilayers respectively. (**a**) Three-dimensional cube of FBAR responses to water, NPA and n-hexane vapors; (**b**) frequency shift for the measurements of water, NPA and n-hexane vapors respectively.

After molecular surface self-assembly of different numbers of PSS/PDDA bilayers on FBAR surface, we compared responses for the measurements of water, NPA and n-hexane vapors. Figure 10a plots the three-dimensional cube of FBAR responses to different vapors with respect to the relative concentration (in terms of $p/p_{0}$) and the number of PSS/PDDA bilayers. The two-dimensional diagram of the cube’s cross section shows the relative concentration or thickness dependent property of the humidity sensor. As shown in Figure 10b, responses to water vapor are higher than NPA and n-hexane vapors with a sequence of water > NPA > n-hexane. This is attributed to the variations of vapor molecule’s polarity property [40]. Water molecules are highly polarized and NPA molecules are moderately polarized, while n-hexane molecules show no polarity property. As a result, polar PSS/PDDA films absorb more water molecules and less NPA molecules, while a negligible amount of n-hexane molecules. Moreover, in contrast to water and NPA vapors, the increment in the bilayer number weakens the sensor signal during N-hexane vapor measurement, as shown in Figure 10b. This is due to the fact that polar PSS/PDDA films act as a passivation layer to prevent the penetration of non-polar n-hexane molecules onto FBAR surface. Additionally, PET-coated FBAR also shows negligible responses to non-polar carbon dioxide (CO_2_), which is a dominating gas under ambient conditions (Supplementary Figure S2). 

It is observed from Figure 10b that the detection limit of the sensor for humidity measurement reaches 0.5% (16.66 ppm), which exceeds the theoretical detection limit (43.64 ppm) of FBAR coated with 30 PSS/PDDA bilayers. This derives from the two-stage manner of FBAR-based humidity sensor: the sensitivity of FBAR coated with 15 PSS/PDDA bilayers is 2068.60 Hz/ppm when the relative concentration is less than 1.5% (55.98 ppm), while it decreases to 127.33 Hz/ppm when the relative concentration ranges from 1.5% (55.98 ppm) to 32% (1194 ppm). This is in accord with the results reported in the literature [30,31,41]. Further investigations are being undertaken with the purpose of a full understanding of the two-stage absorption behavior.

## 4. Conclusions

In summary, we proposed a highly sensitive humidity sensor based on the combination of ultrahigh-frequency microelectromechanical resonator and nano-assembled PET thin films. We first optimized the morphology of PET thin films in order to improve the water molecule absorption efficiency. Compared with tightly-packed nanostructure, loosely-packed nanostructure possesses a larger specific surface area, which provides more absorption sites for water molecules. As a result, the FBAR-based humidity sensor was extremely sensitive with good linearity, stability, reversibility and low detection limit. Since the partial partition coefficient of water vapor in absorption equilibrium increases when the environmental temperature decreases, PET-coated FBAR possesses a higher sensitivity at low temperature. Furthermore, we analyzed the selective detection of water vapor from VOCs with respect to the polarity property. Owing to the high sensitivity, miniaturized size and ultrahigh operating frequency, FBAR coated with PET thin films is found to be a potential candidate as a wireless humidity sensor to realize the integration with WSNs.

## Figures and Tables

**Table 1:** Preparation of polyelectrolyte (PET) thin films with different morphology ^1^.

Sample	Assembly Methods	PET Concentration
**1**	Dipping-assisted	0.2 mg/mL
**2**	Dipping-assisted	20 mg/mL
**3**	Spinning-assisted	20 mg/mL

^1^ FBAR frequency shift was recorded to monitor the total mass of PETs up to the same amount for each sample.

**Table 2:** Sensitivity and linearity of FBARs coated with various number of PSS/PDDA bilayers with the volume concentration of water vapor changing from 373 ppm to 1866 ppm.

Number of Bilayers	Sensitivity (Hz/ppm)	*R*-Square
5	143.61	0.97625
10	345.91	0.99695
15	500.53	0.99576
20	886.11	0.99892
25	1317.20	0.98583
30	2202.20	0.98989

## Supplementary Materials

The following are available online at www.mdpi.com/2072-666X/8/4/116/s1, Figure S1: Fitting results of specific surface area using the BET equation, Table S1: Extracted value of A, B, $\nu_{m}$ and $S_{\mathrm{BET}}$ using the BET equation, Figure S2: Comparison of frequency shift between CO_2_ and water vapor with respect to the various volume concentration.
