# Correction: Liu, W. *et al*. A Highly Sensitive Humidity Sensor Based on Ultrahigh-Frequency Microelectromechanical Resonator Coated with Nano-Assembled Polyelectrolyte Thin Films. *Micromachines*, 2017, *8*, 116

**DOI:** 10.3390/mi8060178

**Published:** 2017-06-05

**Authors:** Wenpeng Liu, Hemi Qu, Jizhou Hu, Wei Pang, Hao Zhang, Xuexin Duan

**Affiliations:** State Key Laboratory of Precision Measuring Technology & Instruments, Tianjin University, Tianjin 300072, China; liuwenpeng@tju.edu.cn (W.L.); mrhjz1222@tju.edu.cn (J.H.); weipang@tju.edu.cn (W.P.); haozhang@tju.edu.cn (H.Z.)

In the published paper [1], there is an error in Figure 3. The red curve in Figure 3b was deleted by mistake during the revisions. The correct figure should read as follows:

The authors apologize for any inconvenience caused by the error. The manuscript will be updated online and the previous version will remain available on the article webpage.

## Figures and Tables

**Figure 3:**
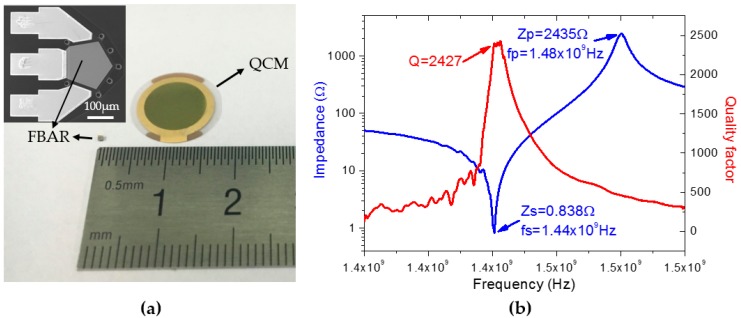
FBAR characterization. (**a**) A comparison of the size between FBAR and quartz crystal microbalance (QCM). The top-left inset shows the top-view scanning electron microscopy (SEM) image of FBAR; (**b**) magnitude of impedance and *Q* value over frequency.
