# Supplementary material for: A Highly Sensitive Humidity Sensor Based on Ultrahigh-Frequency Microelectromechanical Resonator Coated with Nano-Assembled Polyelectrolyte Thin Films
Source: Micromachines (Basel). 2017 Apr 5;8(4):116. doi: 10.3390/mi8040116 (PMC6189836; doi:10.3390/mi8040116)
Supplement: Supplementary file 1 [file micromachines-08-00116-s001.pdf]

Supplementary Materials: A Highly Sensitive Humidity Sensor Based on Ultrahigh-Frequency Microelectromechanical Resonator Coated with Nano-Assembled Polyelectrolyte Thin Films

Wenpeng Liu, Hemi Qu, Jizhou Hu, Wei Pang, Hao Zhang, and Xuexin Duan

1. Calculation of Specific Surface Area (S_{BET})

The specific surface area (S_{BET}) is calculated with the following equations:

$$\frac{1}{v\left(\frac{p_0}{p}-1\right)} = \frac{c-1}{v_{\mathrm{m}}c} \times \frac{p}{p_0} + \frac{1}{v_{\mathrm{m}}c} \tag{S1}$$

$$S_{\mathrm{BET}} = \frac{v_{\mathrm{m}}Ns}{Va} \tag{S2}$$

where p represents the partial vapor pressure and p_0 represents the saturated vapor pressure, v is the absorbed mass of vapor, v_{m} is the monolayer absorbed mass of vapor, c is the BET constant, N is Avogadro's number (6.02×10^{23} mol^{-1}), s is the adsorption cross section of the water molecule ($\pi \times 1.2^2 \times 10^{-20}$ m^2), V is the molar volume of the vapor (22.4 L/mol), and a is the mass of the sensitive layer (2.6×10^{-10} g). To simplify the fitting process, Equation (S1) takes the form:

$$\frac{1}{v} = A\left(1-\frac{p}{p_0}\right) + B\left(\frac{p_0}{p}-1\right) \tag{S3}$$

where

$$A = \frac{c-1}{v_{\mathrm{m}}c} \tag{S4}$$

$$B = \frac{1}{v_{\mathrm{m}}c} \tag{S5}$$

v is calculated by the frequency shift according to Sauerbrey's equation:

$$\frac{\Delta f}{f_s} \approx -\frac{\rho_{\mathrm{m}}d_{\mathrm{m}}}{\rho_0 d_0} \tag{S6}$$

where Δf is the frequency shif ρ_{m} and ρ_0 are the density of the mass loading layer and the resonator layer, d_{m} and d_0 are the thickness of the mass loading layer and the resonator respectively.

Figure S1 and Table S1 show the fitting results whereby the specific surface area is obtained.

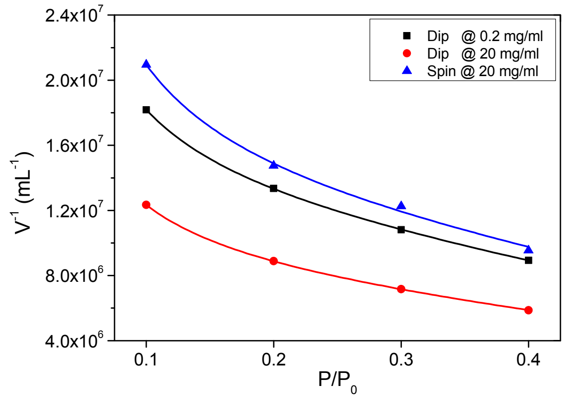

Figure S1. Fitting results of specific surface area using the Brunauer–Emmett–Teller (BET) equation.

Table S1. Extracted value of A, B, v_m and S_{BET} using the Brunauer–Emmett–Teller (BET) equation.

Sample	A	B	v_m	S_{BET}
Dip @ 0.2 mg/mL	1.31×10^7	7.10×10^5	7.24×10^{-8}	3.38×10^2
Dip @ 20 mg/mL	8.49×10^6	5.22×10^5	1.10946×10^{-7}	5.19×10^2
Spin @ 20 mg/mL	1.39×10^7	9.33×10^5	6.72347×10^{-8}	3.14×10^2

2. Responses to Carbon Dioxide (CO_2)

As a dominating gas in the environment, CO_2 causes cross-sensitivity which is a crucial issue for the sensor in real application. In order to determine the cross-sensitivity to CO_2 of polyelectrolyte (PET)-coated film bulk acoustic resonator (FBAR), we recorded responses to CO_2 via FBAR coated with 30 poly(sodium 4-styrenesulfonate)/poly(diallyldimethytlammonium choride) (PSS/PDDA) bilayers. Compared with water vapor, PET-coated FBAR shows negligible responses to CO_2, as shown in Figure S2. This is attributed to the fact that polar PSS/PDDA films absorb negligible amount of non-polar CO_2.

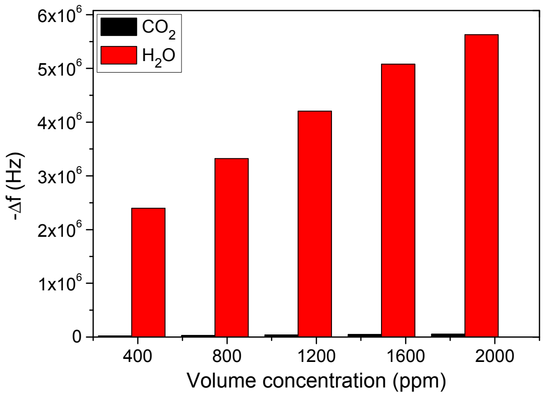

Figure S2. Comparison of frequency shift between CO_2 and water vapor with respect to the various volume concentration.
